# *Listeria* Rhombencephalitis Complicating Anti-TNF Treatment during an Acute Flare of Crohn's Colitis

**DOI:** 10.1155/2016/6216128

**Published:** 2016-08-29

**Authors:** L. Stratton, G. R. Caddy

**Affiliations:** ^1^Belfast City Hospital, Lisburn Road, Belfast, UK; ^2^Ulster Hospital Dundonald, Upper Newtownards Road, Belfast, UK

## Abstract

Patients with Crohn's disease often require the use of immunosuppressant drugs to control disease activity. Such medication includes steroids, azathioprine, and biologic therapy. These suppress the immune response, and the patient is more susceptible to infection. We present a case of a 69-year-old gentleman with a history of Crohn's colitis who had ongoing symptoms of diarrhoea in spite of standard treatment. Biologic therapy was considered to be the next step, and screening for infection was undertaken prior to use. Three days following anti-TNF treatment, he became drowsy, and examination revealed pyrexia, slurred speech, and nystagmus. Investigation revealed presence of* Listeria* rhombencephalitis. He demonstrated poor neurological recovery.* Listeria monocytogenes* is an infection commonly associated with food sources. Some patients develop a self-limiting diarrhoeal illness, but in the immunosuppressed population, the clinical features may be more sinister. Cotrimoxazole prophylaxis is already recommended for those on triple immunosuppression. We propose the early initiation of this treatment, including where biologic use is anticipated. In those on multiple immunosuppressants, a diet similar to that followed in pregnancy may minimise risk of acquiring this infection. Clinicians must always have a high index of suspicion for opportunistic infection in such immunocompromised patients.

## 1. Introduction

Crohn's disease is an inflammatory condition that predominantly affects the gastrointestinal tract. Immunosuppression is the mainstay of treatment for the disease, and conventional induction of remission involves the use of steroids. Maintenance of remission should then involve introduction of an agent such as azathioprine, mercaptopurine, or methotrexate [1]. For those in whom remission cannot be achieved by such agents, escalation to biologic therapy with anti-TNF-alpha medication should be considered [1].

Infliximab is a potent immunosuppressant and patients must be screened for infection prior to introduction of the agent. The European Crohn's and Colitis (ECCO) guidelines state that all patients should be screened for evidence of latent tuberculosis (TB), hepatitis B and C virus (HBV and HCV), and HIV. Patients should also be screened for previous Varicella Zoster Virus (VZV) infection. Screening for CMV and EBV is not routinely recommended but may be considered in certain cases [2]. Use of cotrimoxazole prophylaxis against* Pneumocystis jiroveci* is recommended if patients are prescribed triple immunosuppression including either a calcineurin inhibitor or a TNF-alpha inhibitor [2]. In cases of dual immunosuppression, prophylaxis should still be considered, particularly with use of calcineurin inhibitor [2].

## 2. Case

A 69-year-old man was admitted to a district general hospital with a flare of Crohn's colitis. His symptoms included bloody diarrhoea, with ten to fifteen episodes per day. He had a two-year history of ileocolonic Crohn's disease, and otherwise his past medical history was unremarkable. At ward level, he was alert, orientated, and independent with personal self-care.

Conventional treatment with 5-ASAs and azathioprine had failed to achieve remission, although he had been unable to tolerate doses greater than 100 mg of azathioprine due to nausea. As a result, he had become steroid dependent and had been taking continuous prednisolone for seven months prior to admission, with 20 mg being the lowest dose achieved during that time. Intravenous steroids (100 mg hydrocortisone qds) did not improve symptoms when he was admitted to hospital, and as per the initial outpatient plan discussed with the surgical team, biologic therapy was considered to be the next step for this gentleman. At the time of admission, he had suffered recurrent episodes of pyrexia. Three sets of peripheral blood cultures showed no growth and neither did serial stool cultures. Plain chest radiograph was unremarkable.

He was counselled and commenced on Infliximab treatment. Three days later, he suffered a clinical deterioration, with drowsiness and pyrexia. Glasgow Coma Scale revealed that eyes opened to voice, speech was limited to words only, not sentences, and he was able to obey commands (GCS 12/15). Full neurological examination was inhibited by patient drowsiness. All four limbs moved symmetrically against gravity, sensation appeared intact, and reflexes were equal and symmetrical. Limited cooperation inhibited assessment of limb coordination but horizontal nystagmus was noted in both right and left lateral gaze and speech was slurred. He had no rash or neck stiffness. A new cold sore was noted on his top lip.

Urgent blood tests were sent alongside further blood, stool, and urine cultures. He underwent CT brain that day which showed no acute intracranial abnormality. He then underwent lumbar puncture. Results of these investigations are shown in Table 1. At the point of deterioration, he had been commenced empirically on acyclovir and high dose meropenem (2 g tds) to cross the blood-brain barrier.

Prior to confirmation of the organism, lumbar puncture had revealed high protein and low glucose in cerebrospinal fluid (CSF), and he was commenced empirically on isoniazid, rifampicin, ethambutol, and pyrazinamide to cover for tuberculosis. These were stopped when* Listeria* was found in both peripheral blood cultures and CSF. It was shown to be sensitive to meropenem but antibiotics were changed to gentamicin and high dose amoxicillin (2 g qds) on the advice of Microbiology. They suggested a six-week course of amoxicillin and up to three weeks of gentamicin.

MRI imaging revealed extensive T2 and FLAIR high signal centred on the cerebellar vermis with extension into the cerebellar hemispheres and brainstem, in keeping with rhombencephalitis.

He was transferred to the Intensive Care Unit (ICU) five days following Infliximab infusion due to fluctuating GCS in the range of 3–10/15 and was intubated in ICU because of concerns regarding airway protection. He transferred to ICU in the tertiary referral centre for neurology and neurosurgery for closer input, as he was deemed to be at high risk of developing obstructive hydrocephalus. In ICU, he underwent tracheostomy and was later weaned from mechanical ventilation. He also underwent gastrostomy insertion for long term nutrition maintenance.

Subsequent neurological evaluation revealed prominent spastic quadriparesis with occasional spontaneous nonpurposeful movement in his upper limbs and a relatively fixed reduction of consciousness, with no evidence of awareness. He spent one month in ICU and was then discharged to ward level care. Active Crohn's disease was an ongoing problem, although medication was limited to steroids, and he was deemed to be too unwell to undergo defunctioning bowel surgery. No evidence of neurological recovery was demonstrated during the months that followed and he suffered from recurrent episodes of aspiration pneumonia. The latter was listed as primary cause of death ten months following Infliximab infusion, with* Listeria* rhombencephalitis and Crohn's disease listed as contributory factors.

Prior to Infliximab treatment, this gentleman was already immunosuppressed. Long term steroid use alongside azathioprine already rendered him at risk of developing opportunistic infection. However, the serially negative blood cultures until the point of administration, followed by positive blood and CSF cultures 3 days following Infliximab, would suggest that this did play a role in his deterioration. This too is suggested by his dramatic neurological deterioration so soon after infusion.

## 3. Discussion

*Listeria monocytogenes* is a gram positive bacillus [3]. Its most common mode of transmission is by the ingestion of contaminated food, thought to occur in about 99% of reported infections [4]. Foods of particular risk include soft cheese, unpasteurised dairy products, cold meats, and prepackaged foodstuffs including sandwiches [5]. Unlike other common food pathogens, it is able to replicate at temperatures as low as 4°C often found in refrigerators. The pathogen is killed by heating and by pasteurisation [6].

*Listeria* infection is associated with an approximately 20–30% mortality rate [7]. Those at particular risk include pregnant women, patients above the age of 75, and patients who are immunocompromised [8]. It is also worth noting that patients who regularly take acid-lowering medication, such as proton pump inhibitors, may be at higher risk. The acid conditions usually found in the stomach confer a degree of protection against the bacterium but alkalinisation may reduce the natural barrier to such pathogens [4].

*Listeria* infection can present in a number of different ways. Often, the main feature is one of a diarrhoeal illness that is self-limiting. It is unclear which patients will progress to more severe presentations, such as with central nervous system (CNS) involvement. CNS sequelae include meningitis, cerebritis, abscesses, and rhombencephalitis [6]. Complications of CNS syndromes include seizures and hydrocephalus, and these carry a high mortality rate. CNS involvement is an independent risk factor for mortality [5].

A study examining a number of outbreaks of* Listeria* demonstrates a range of incubation periods which appear to be associated with different features of disease [6]. Patients who developed* Listeria* infection in pregnancy had a significantly longer period of incubation (median 27.5 days, range 17–67) before clinical manifestations of the disease occurred. Those with CNS involvement had a median of 9 days of incubation before symptoms. Those presenting with bacteraemia had the shortest incubation period with a median of 2 days [6].

Often patients who take multiple immunosuppressive agents on a long term basis are prescribed cotrimoxazole for the prevention of* Pneumocystis jiroveci* pneumonia. It is recognised that the use of cotrimoxazole may also be of use in the prophylaxis against* Listeria*,* Legionella*, and* Toxoplasma* [9]. The use of such prophylaxis could potentially reduce the number of cases of* Listeria* in high risk individuals.

All immunosuppressants, including steroids, leave patients at risk of opportunistic infection by dampening the body's natural immune response. A study found that 36% of patients taking Infliximab will develop an infection each year. This study, of more than 6000 patients, found that patients taking Infliximab were at greater risk of developing potentially life-threatening infections, and this risk remained when adjusted for steroid use [10].

Alongside risk conferred by multiple immunosuppressants, this gentleman was also 69-year-old at initiation of biologic therapy. A study of 100 consecutive patients with IBD and an opportunistic infection examined the risk factors for development of such an infection. Risk was significantly increased in the population over 50 compared with those under 25 [11]. This study also demonstrated that patients are at higher risk if they are taking multiple medications to suppress immune response [11].

## 4. Conclusion

Overall, it is known that those on anti-TNF medications are at higher risk of opportunistic infections and that* Listeria* tends to follow a more aggressive course in these patients. We propose that they be counselled on high risk foodstuffs prior to treatment, alongside preparation and cooking techniques for such food-borne infections [12]. Similar advice to that given to pregnant women may be useful. Early initiation of prophylactic cotrimoxazole should be used and could potentially be commenced during the screening process for biologic therapy. Any clinical deterioration in these patients should be accompanied by a high index of suspicion for opportunistic infection, given their immunocompromised state. Caution must always be exercised in the elderly population who already have a high risk of opportunistic infection.

## Figures and Tables

**Table 1:** Laboratory values for blood and cerebrospinal fluid (CSF) tests.

Hb	121 g/L
WCC	8.6 × 10^9^/L
Plt	405 × 10^9^/L
Bili	19 *μ*mol/L
ALP	48 U/L
AST	14 U/L
GGT	74 U/L
Alb	29 g/L
Na^+^	136 mmol/L
K^+^	3.8 mmol/L
Urea	4.6 mmol/L
Creat	56 *μ*mol/L
HCO_3_^−^	21 mmol/L
Glucose	8.5 mmol/L
Lactate	1.9 mmol/L
Ammonia	22 *μ*mol/L
CRP	52 mg/L
*C*. Ca^2+^	2.32 mmol/L
PO_4_^3−^	0.76 mmol/L

*CSF analysis*	
Glucose	1.1 mmol/L
Protein	0.77 g/L
RBC	0/mm^3^
WCC	40/mm^3^
Gram stain	Nil seen
Culture	*Listeria monocytogenes*
